# First ovum-in-ovo pathological titanosaurid egg throws light on the reproductive biology of sauropod dinosaurs

**DOI:** 10.1038/s41598-022-13257-3

**Published:** 2022-06-07

**Authors:** Harsha Dhiman, Vishal Verma, Guntupalli V. R. Prasad

**Affiliations:** 1 Department of Geology, Center for Advanced Studies, University of Delhi, Delhi, 110007 India; 2 Higher Secondary School, Dhar District, Bakaner, Madhya Pradesh India

**Keywords:** Geology, Palaeontology

## Abstract

Pathologic eggs have been documented in the amniote eggs of birds, turtles, and dinosaurs. These eggs occur either in the form of one egg within another egg, a condition known as ovum-in-ovo or multi-shelled eggs showing additional pathological eggshell layer/s besides the primary shell layer. Though multi-shelled eggs and eggshells were previously recorded only  in reptiles and ovum-in-ovo eggs in birds, now it has been shown that multi-shelled egg pathology occurs in birds as well. However, no ovum-in-ovo egg has been reported  in dinosaurs or for that matter  in other reptiles. Here we describe an ovum-in-ovo pathological egg from a titanosaurid dinosaur nest from the Upper Cretaceous Lameta Formation of western Central India which makes it the first report of this pathology in dinosaurs. Birds possess a specialized uterus while other amniotes have a generalized uterus. However, alligators and crocodiles retain a specialized uterus like birds along with a reptilian mode of egg-laying. The discovery of ovum-in-ovo egg from a titanosaurid dinosaur nest suggests that their oviduct morphology was similar to that of birds opening up the possibility for sequential laying of eggs in this group of sauropod dinosaurs. This new find underscores that the ovum-in-ovo pathology is not unique to birds and sauropods share a reproductive behavior very similar to that of other archosaurs.

## Introduction

Two traits that collectively characterize dinosaurs, birds, and other archosaurs are oviparous mode of reproduction and cledoic egg, the latter of which was instrumental in the rise of amniotes^1^. These cledoic eggs are notably observed in the oological records of theropods, sauropodomorphs, and ornithischians more or less from all over the globe^2–5^, along with the rare occurrences of embryos^6–8^. These oological records have helped in analyzing dinosaur reproduction and nesting behavior which fall between the other archosaurs, such as crocodiles and birds. Different egg macroscopic features and eggshell micro-structures occur in different types of dinosaurs, such as, spherical-shaped eggs in sauropods and elliptical-shaped eggs in theropods^6,7^. Moreover, bird-like behavior has been observed in the fossil record of dinosaurs in the form of nest constructions and evidences of parental care especially in derived theropods^9,10^, while other dinosaurs, such as hadrosaurs and sauropods, spent their time in building communal nests and excavated bowl-shaped clutches^5,11^. The bird-like incubating behavior has been inferred from the fossilized nests of oviraptorids^6^, which is not observed in sauropods and hadrosaurs, who possibly achieved incubation through sediment burial^12^. These observations along with phylogenetic analyses have suggested that bird-like features appeared in derived theropods much before the evolution of birds^13,14^, while sauropods show reproductive traits similar to crocodiles^13^, notably the presence of random piles of clutches that would have resulted from the presence of two oviducts^15^. Differences also exist in eggshell radial structure of these dinosaur groups. For example, sauropod eggshells exhibit a single layer of calcitic crystals while the eggshells of troodontids and oviraptorosaurids are two or three layered^16,17^, while birds have a single functional oviduct and three layered eggshell units^15^.

**Figure 1:**
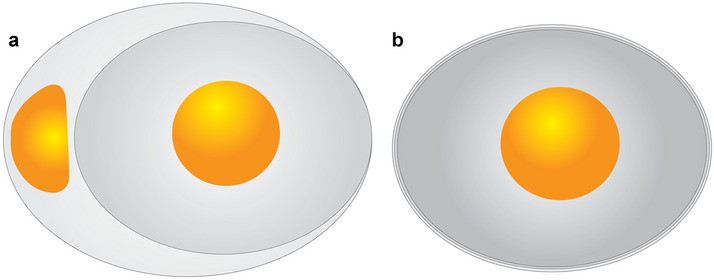
Pathologic cledoic eggs showing unusual morphology not similar to the  normal eggs. (**a**) Ovum-in-ovo pathologic egg characterized by an egg within an egg or two yolks. (**b**) Multi-shelled egg distinctive of two or more than two eggshell layers surrounding an individual egg (modified after Carpenter^3^, software used Adobe Illustrator CS6).

Another important aspect noticed in the physical and microstructural features of reptilian and bird eggs and eggshells is pathology. Abnormal or pathologic eggs consist of unusually large or small-sized eggs, egg within another egg, eggs without yolk, and abnormally shaped eggs. The abnormalities reflected in the eggshells include multiple eggshell units occurring in close contact with each other and one above the other (multi-shelled), abnormally thick or thin eggshells, abnormally shaped shell units, extra shell units blocking pore canals, and surface defects^3^. The two important pathologies that help in understanding reproductive behavior of amniotes are ovum-in-ovo (Fig. 1a) and multi-shelled egg (Fig. 1b)^3^. An ovum-in-ovo egg of modern birds consists of an egg within an egg with a distinct gap between the two eggshell layers which is occupied by the yolk^18–22^. Multi-shelled eggshells are distinct from the ovum-in-ovo eggs of birds in having closely spaced calcitic eggshell layers one above the other with or without an organic membrane in between^3^. Multi-shelled eggs are abnormally thick composing of one primary eggshell layer and one or more pathological layers which occur external to the primary layer. The multi-shelled egg pathology has so far been reported from the eggs of turtles^23–27^, dinosaurs^28–33^, an unidentified crocodilian eggshell^23,29,31^ and lizards^28^. Although in the past, multi-shelled eggs were considered characteristic of reptiles, in recent years, they have also been reported from the eggs of hen^22,34,35^, herring gull^36^, Japanese quail^29^, tropical mockingbird^37^, and a fossil enantiornithine bird^38^. It has been cautioned that one should be careful while considering isolated multi-layered eggshells (not observed in a complete egg or eggs of a nest) as taphonomic processes of crushing and overlapping of eggshells may place them one above the other reminiscing those formed due to normal biological processes^27,39^. In the past, some multi-shelled eggs of turtles and dinosaurs have been identified with supposed ovum-in-ovo pathology^23,30^. Later it became clear that all those dinosaur eggshells identified as representing ovum-in-ovo pathology actually represent multi-shelled condition and ovum-in-ovo pathology occurs only in birds^3,18–22^.

**Figure 2:**
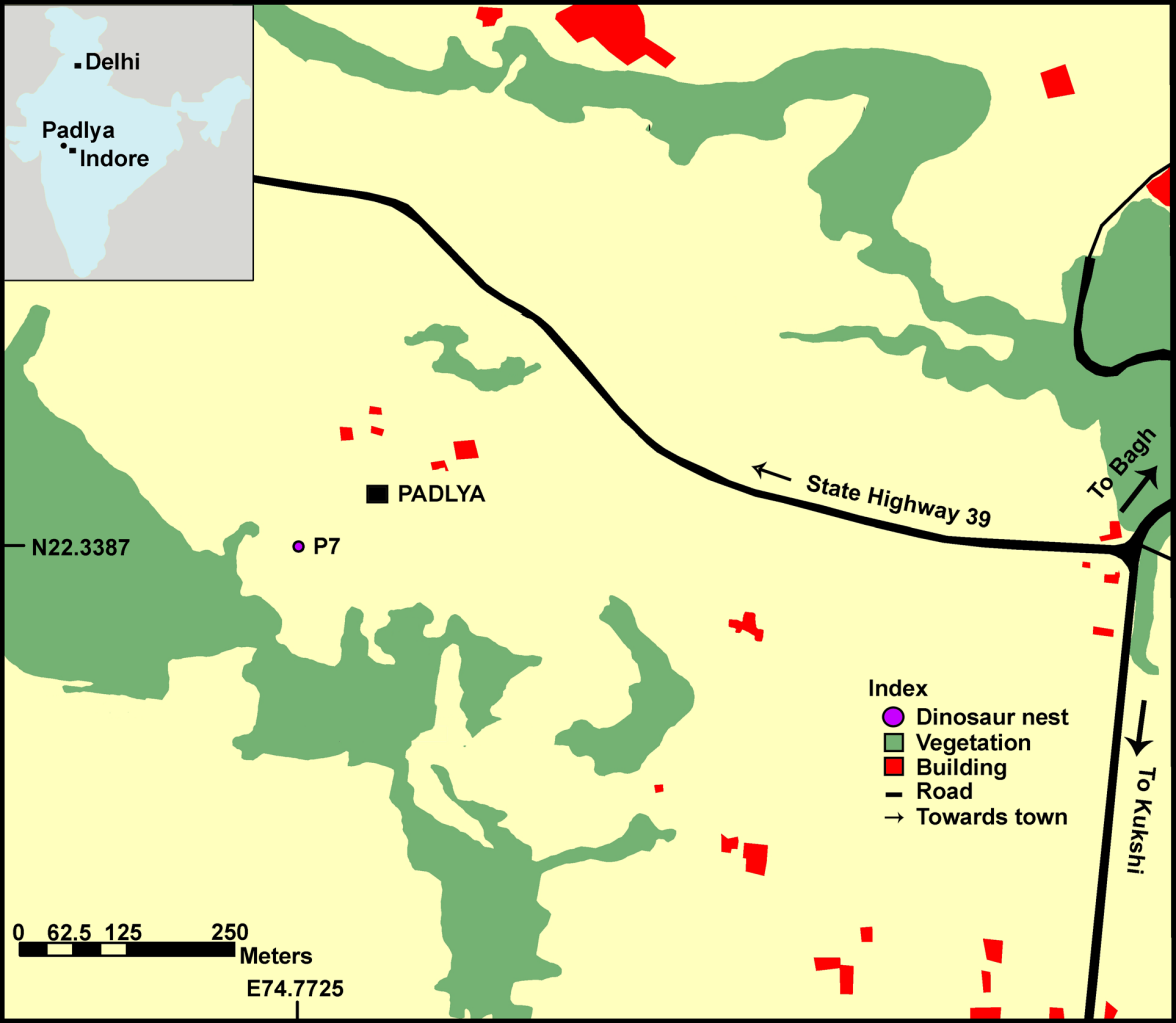
Location map of the study area showing pathologic dinosaur nest P7 from the village Padlya, Dhar District, Madhya Pradesh, India (software used Adobe Illustrator CS6).

So far there has been no report of ovum-in-ovo egg pathology in dinosaur eggs. Considering that the derived theropods share reproductive features similar to birds, this pathology  might have existed in  dinosaurs^3^. During the Ph.D. field work of the senior author (Harsha Dhiman), an unusually double-layered in-situ egg was found in the Upper Cretaceous Lameta Formation of Padlya of Dhar District, Madhya Pradesh (M.P.) state, western Central India (Fig. 2) in a titanosaurid sauropod nest. The cross-sectional outline of the pathologic egg shows physical organization of the two shell layers similar to the ovum-in-ovo eggs previously reported in birds. In this paper, we describe the newly found ovum-in-ovo pathological titanosaurid egg and discuss its preservation and relevance for understanding sauropod reproductive biology. This find represents the first discovery of ovum-in-ovo pathology in dinosaurs. We hypothesize that ovum-in-ovo pathology is not unique only to birds, and sauropod dinosaurs had a reproductive anatomy more similar to those of archosaurs such as crocodiles and birds, rather than to those of non-archosaurian reptiles like turtles and lizards. Throughout the text, the use of the term eggshell layer is with respect to the calcitic shell units and does not refer to the structural layers (e.g., mammillary, continuous, external layers) of the eggshell.

## Geological set-up of the pathological egg-bearing horizon

The in-situ pathological titanosaurid dinosaur egg was found in the sandy limestone/calcareous sandstone of the Upper Cretaceous Lameta Formation exposed in the lower Narmada valley. The Lameta Formation in the Narmada valley consists of arenaceous, argillaceous, and calcareous sediments deposited in continental palaeoenvironmental conditions in a semi-arid to arid palaeoclimatic setting^40^. It is widely distributed in the form of isolated and patchy outcrops in central (M.P., Maharashtra) and western India (M.P., Gujarat). The Lameta Formation is well known for its titanosaur nesting sites, isolated eggs and eggshell fragments^40^. During multiple field-works conducted by the authors in the Bagh-Kukshi areas of Dhar District of M.P., 108 titanosaur nests were identified which include intact clutches, isolated eggs and several eggshell fragments.

**Figure 3:**
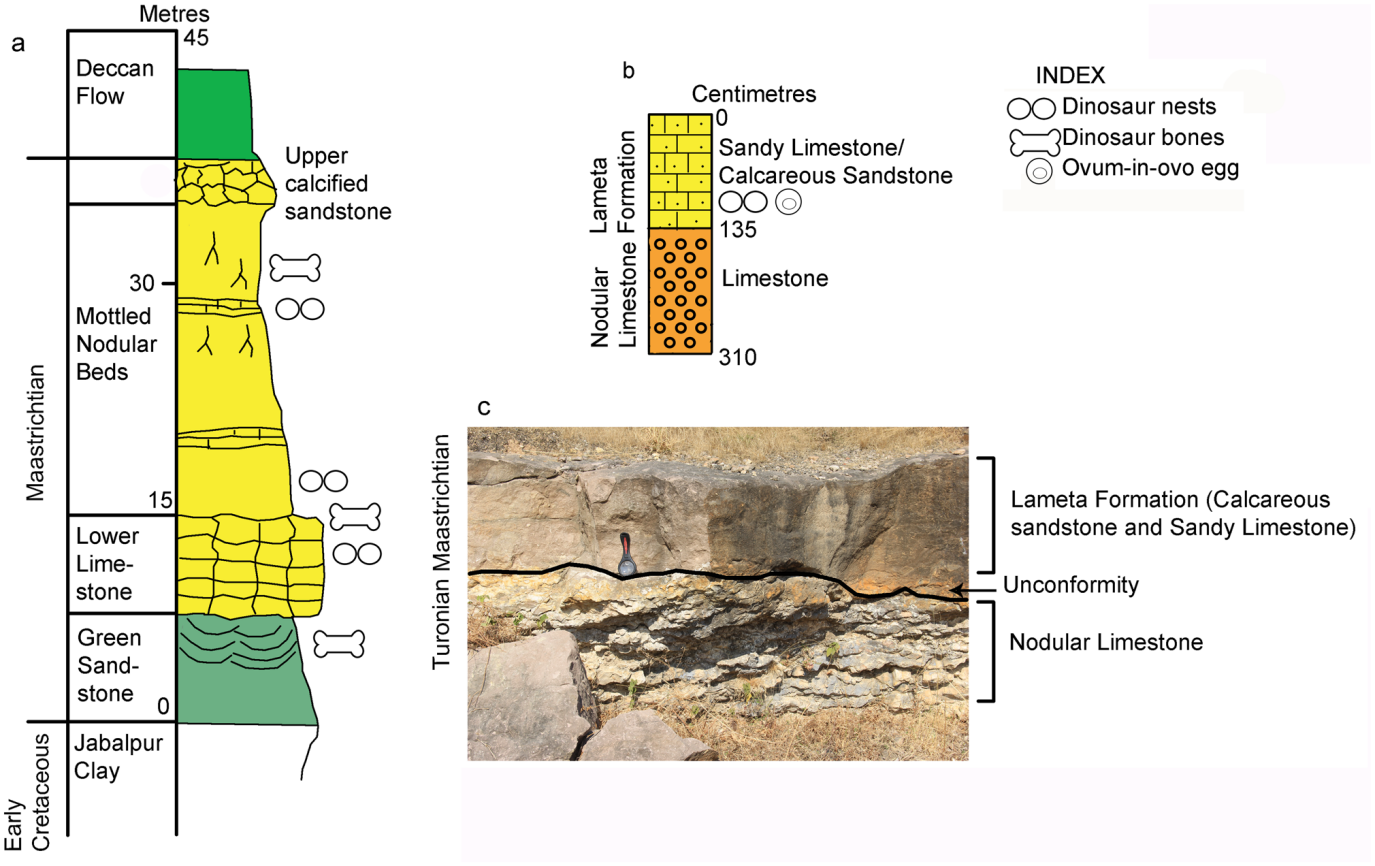
Stratigraphic subdivisions of the Lameta Formation; (**a**) Jabalpur (modified after Tandon et al.^41^); (**b**) Padlya; (**c**) Field photograph showing the  sandy  limestone/calcareous  sandstone of the Lameta Formation overlying the Nodular Limestone of the Bagh Group within Dinosaur Fossil National Park (DFNP), near Padlya, Dhar district, M.P. The Nodular Limestone (Bagh Beds) shows lensoid character while the sandy limestone/calcareous sandstone has massive appearance (**a**,**b** drawn using software Adobe Photoshop CC).

The nest preserving the pathologic egg is documented from the village Padlya of Dhar District, M.P. (Fig. 2) where the Lameta outcrops measure less than a meter to 4–5 m in thickness and exhibit dark-grey surface color. The lithology is enriched in coarse silt-sized to very coarse sand-sized, subangular to subrounded grains of quartz floating in clay-sized carbonate matrix (micrite). This indicates mixed carbonate-siliciclastic lithofacies and we classify the rocks as sandy limestones (showing higher micrite content) and calcareous sandstones (with higher content in clastics). These lithological units also laterally grade into maroon-colored ferruginous sandstone and at places, the latter overlies the former. While the Lameta type section in the Upper Narmada valley is divided into the basal Green Sandstone, the Lower Limestone, the Mottled Nodular Bed, and the Upper Calcareous Sandstone^41^, all these lithological units of the Lameta type section are not preserved in the Lameta outcrops of the present study area except for the possible correlative of the Lower Limestone. Here the Lameta Formation unconformably overlies either the Coralline Limestone or the Nodular Limestone (Fig. 3) of the Upper Cretaceous Bagh Group.

The outcrops from the study area show various sedimentary features, such as mottling, nodules, brecciation, desiccation cracks, reworking of carbonate fragments, and alveolar-septal fabric, and textures consisting of dispersed siliciclastic grains and intraformational clastic grains in micrite-microspar, cracks filled with spar, and microspar crystals around quartz grains. All these features point to palustrine lake deposits that co-existed with alluvial channels and floodplains where maroon-colored sandstones formed and experienced subaerial exposure and oxidation^42–44^.

## Description of Nest P7

Fifty-two sauropod dinosaur nests have been documented from the village Padlya in the form of clutches consisting of two to twenty eggs, nests with single eggs, and outcrops with only eggshell fragments. The pathologic ovum-in-ovo egg has been documented from an outcrop (nest number P7) situated near the Padlya village, which has now been shifted to the premises of Dinosaur Fossil National Park (designated by M.P. state government), close to Padlya village to keep it safe from illegal fossil collectors.

**Figure 4:**
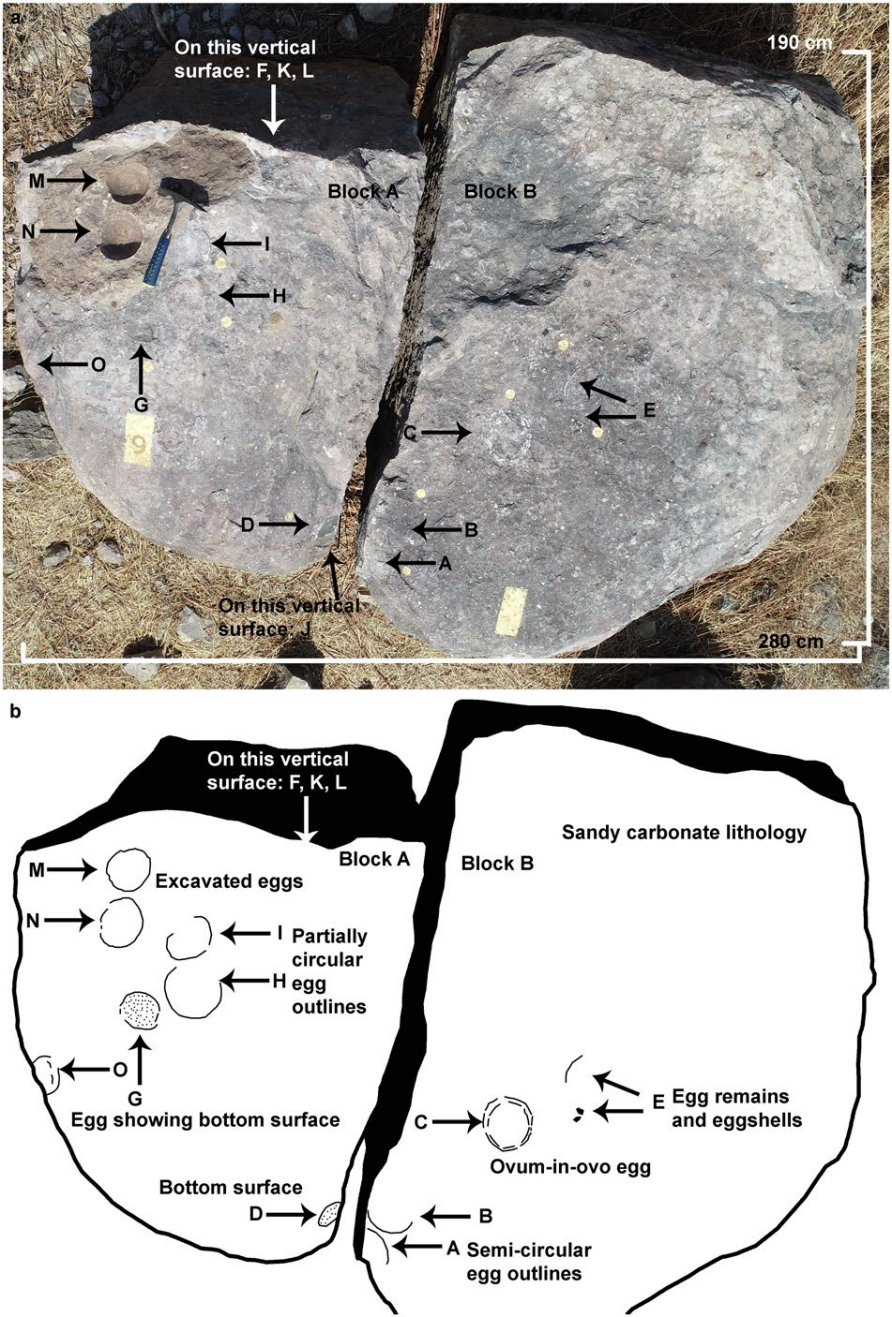
Field photograph and schematic diagram of the outcrop showing nest P7 and its eggs and eggshell fragments belonging to a titanosaur sauropod randomly spaced with respect to each other in a circular nest arrangement. The captions A to O indicate eggs and eggshell locations (Fig. 4b drawn using software Adobe Photoshop CC).

**Figure 5:**
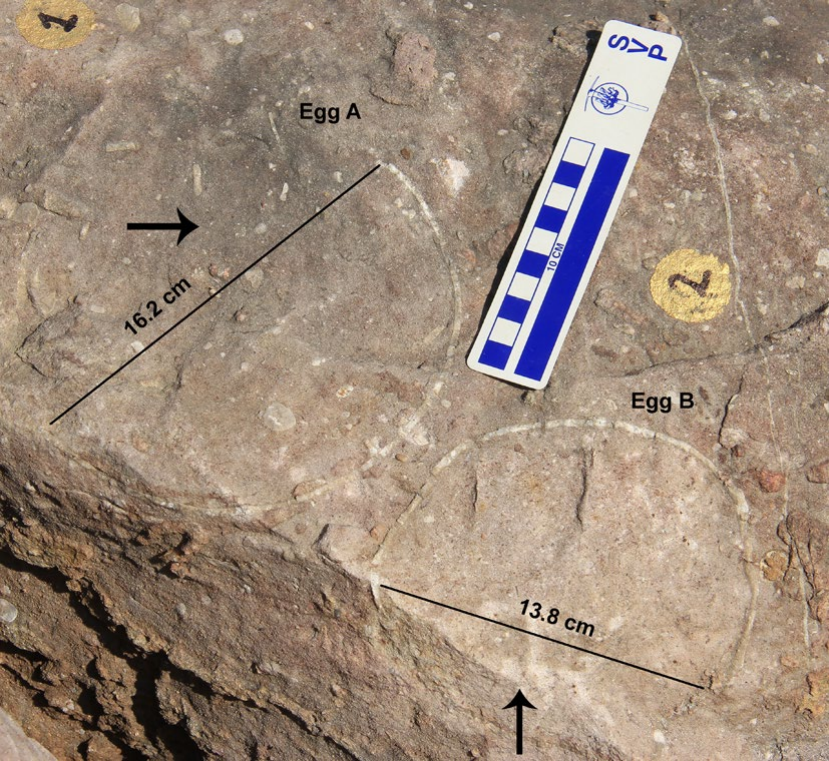
Field photograph of eggs A and B from nest number P7. The eggs exist as semi-circular outlines and the gaps (see arrows) possibly represent hatching windows.

**Figure 6:**
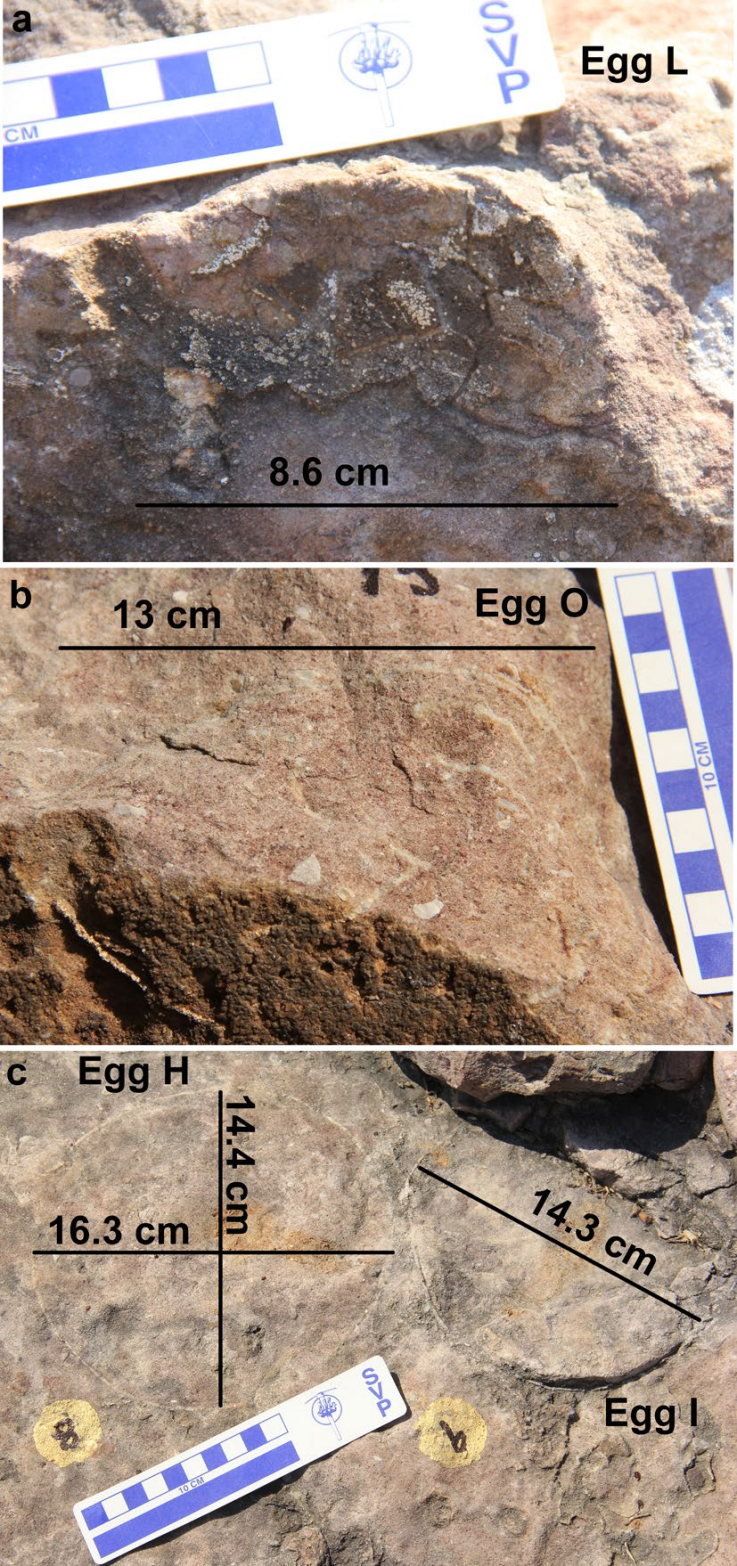
Field photographs of eggs from nest number P7. (**a**) Circular rock with few encrusted eggshells. (**b**) Elliptical  egg with shell fragment pile. (**c**) Circular to semi-circular eggs.

**Figure 7:**
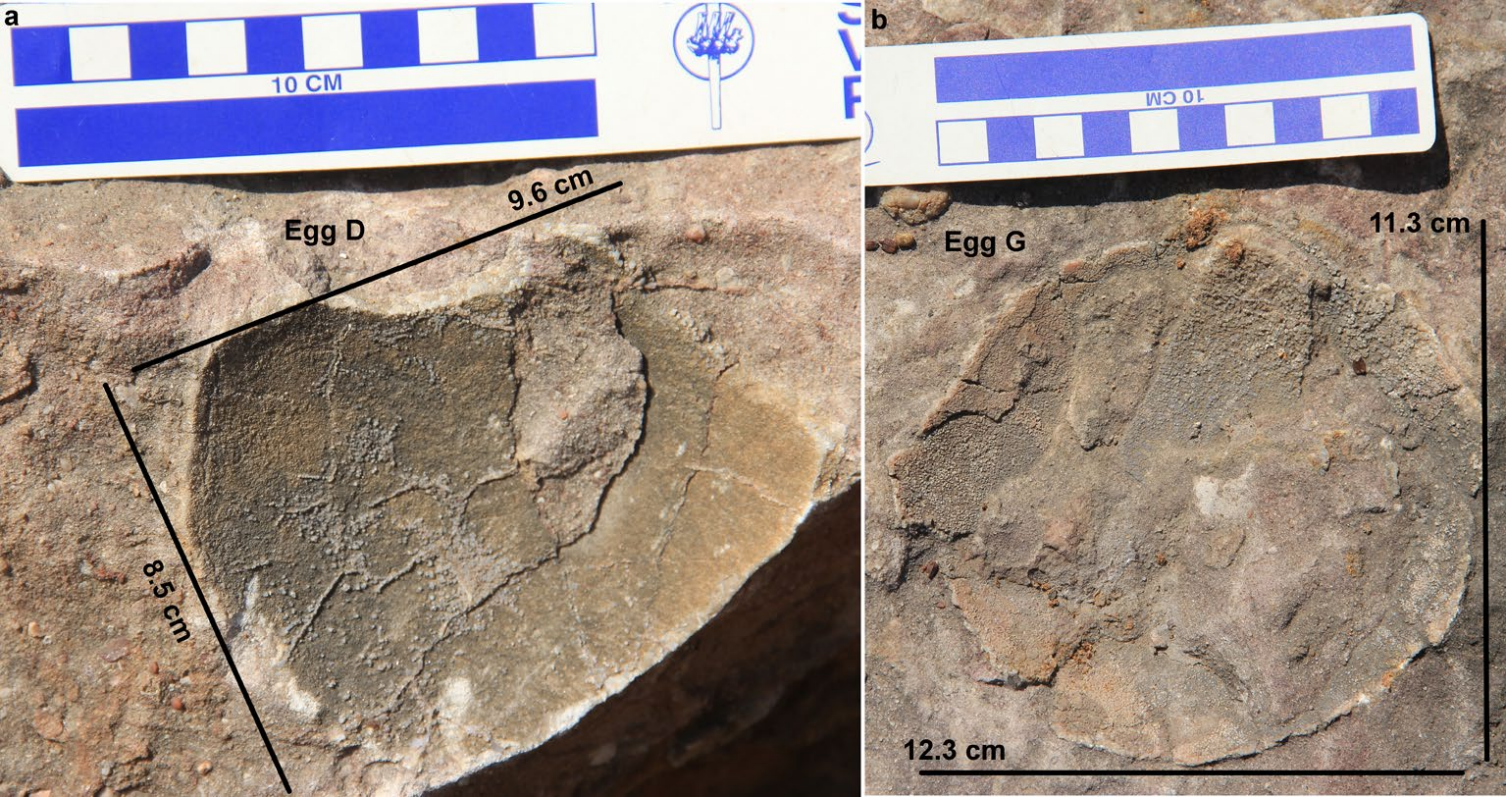
Field photographs of eggs from nest number P7. (**a**) Partially preserved bottom surface of an egg. (**b**) Circular bottom surface of an egg with encrusted eggshells.

**Figure 8:**
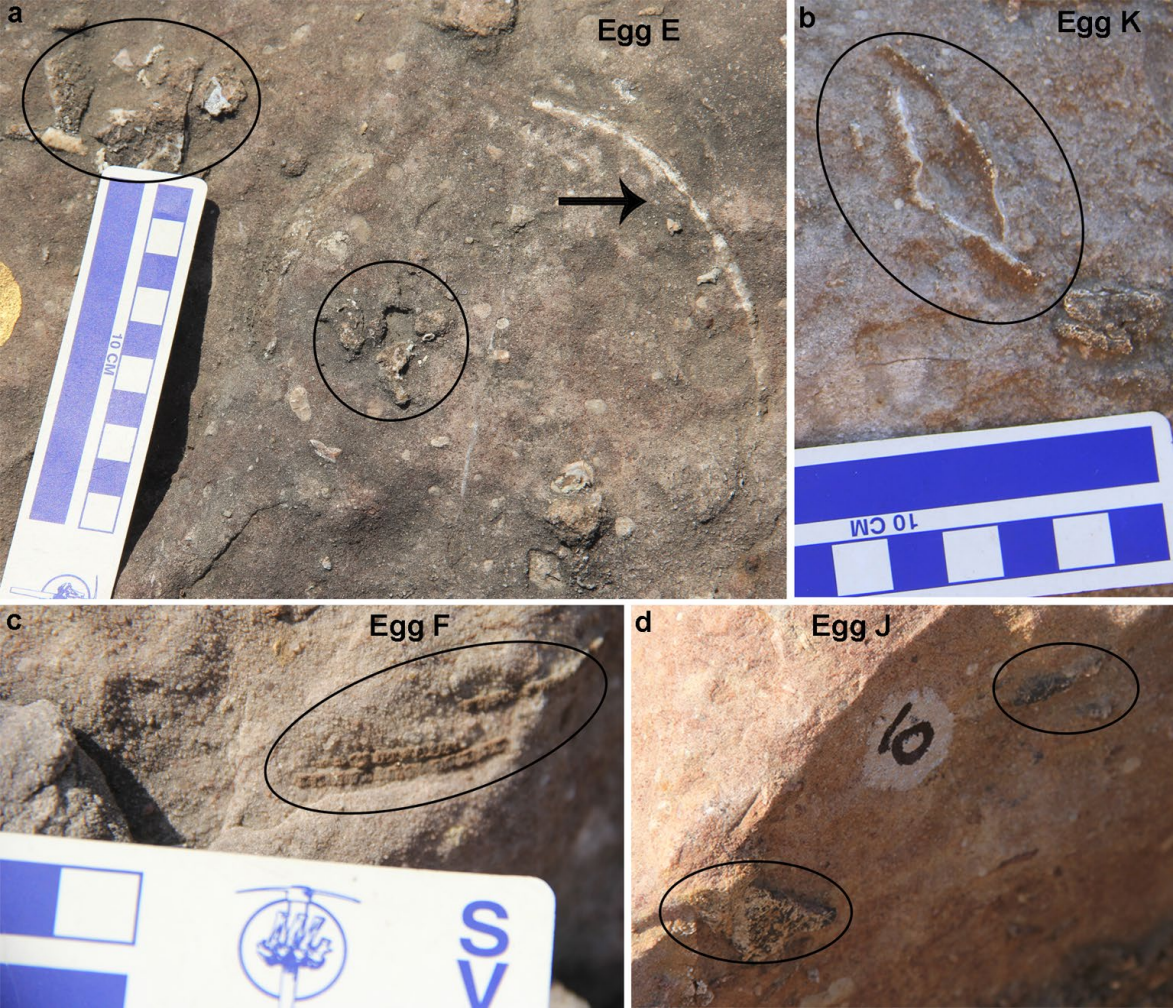
Field photographs of eggs from nest number P7 showing randomly distributed eggshell fragments.

The nest P7 preserves eleven eggs randomly placed with respect to each other in a circular nest arrangement as indicated by variable diameters of circular shaped eggs (Fig. 4). The eggs are present in the form of semi- to completely circular outlines (Figs. 5, 6), bottom surfaces of eggs encrusted with eggshells (Fig. 7), circular-shaped molds of eggs (Fig. 6a), and as eggshell fragments present inside and/or around the egg outlines (Fig. 6b). Some eggshell fragments also occur randomly (Fig. 8). The minimum and maximum egg diameters are 13 cm and 16.6 cm, respectively and the eggs are sub-circular to elliptical in shape, the latter of which is a compressed egg. Generally, megaloolithid eggs belonging to titanosaur sauropods show spherical and sub-spherical shapes with maximum diameter ranging from 12 and 15 cm^13^. Some eggs show a missing upper surface from where the hatchling might have emerged out of the egg (Fig. 5). This is known as hatching window and has also been previously known in titanosaur eggs^45^. This could also be an artefact of sediment erosion. The hatching windows are known to occur on the upper side of the eggs^45,46^. In order to ascertain the upper surface of an egg, it should be found in a vertical stratigraphic section. Since the nest was found in a scattered block of sandy limestone displaced from original outcrop, it is difficult to know the lower or upper stratigraphic surfaces of the block. As the nest is embedded in a huge block, computed tomography analysis could not be done to see the egg in three dimensions and observe any eggshell fragments on the floor of the egg. It is thus difficult to ascertain if these gaps are true hatching windows or a result of sediment erosion. The nest P7 shows an egg with eggshells scattered within its periphery; this is another commonly known feature of titanosaur eggs and is known as shell fragment pile^47^ (Fig. 6b). The random distribution of eggs and their variable diameters suggest circular/clutch type nest arrangement which is a common nest type observed in sauropods^48^. Parataxonomic classification of the eggs from this nest is presented under systematic palaeontology section.

## Systematic palaeontology

Oospecies *Fusioolithus baghensis* (Khosla and Sahni^49^ Fernández and Khosla^50^).

*Megaloolithus baghensis* (Khosla and Sahni^49^).

*Megaloolithus balasinorensis* (Mohabey^51^).

*Stratigraphic horizon and locality* The Upper Cretaceous Lameta Formation exposed near the village Padlya of Dhar District, M.P., India.

*Age* Maastrichtian.

**Figure 9:**
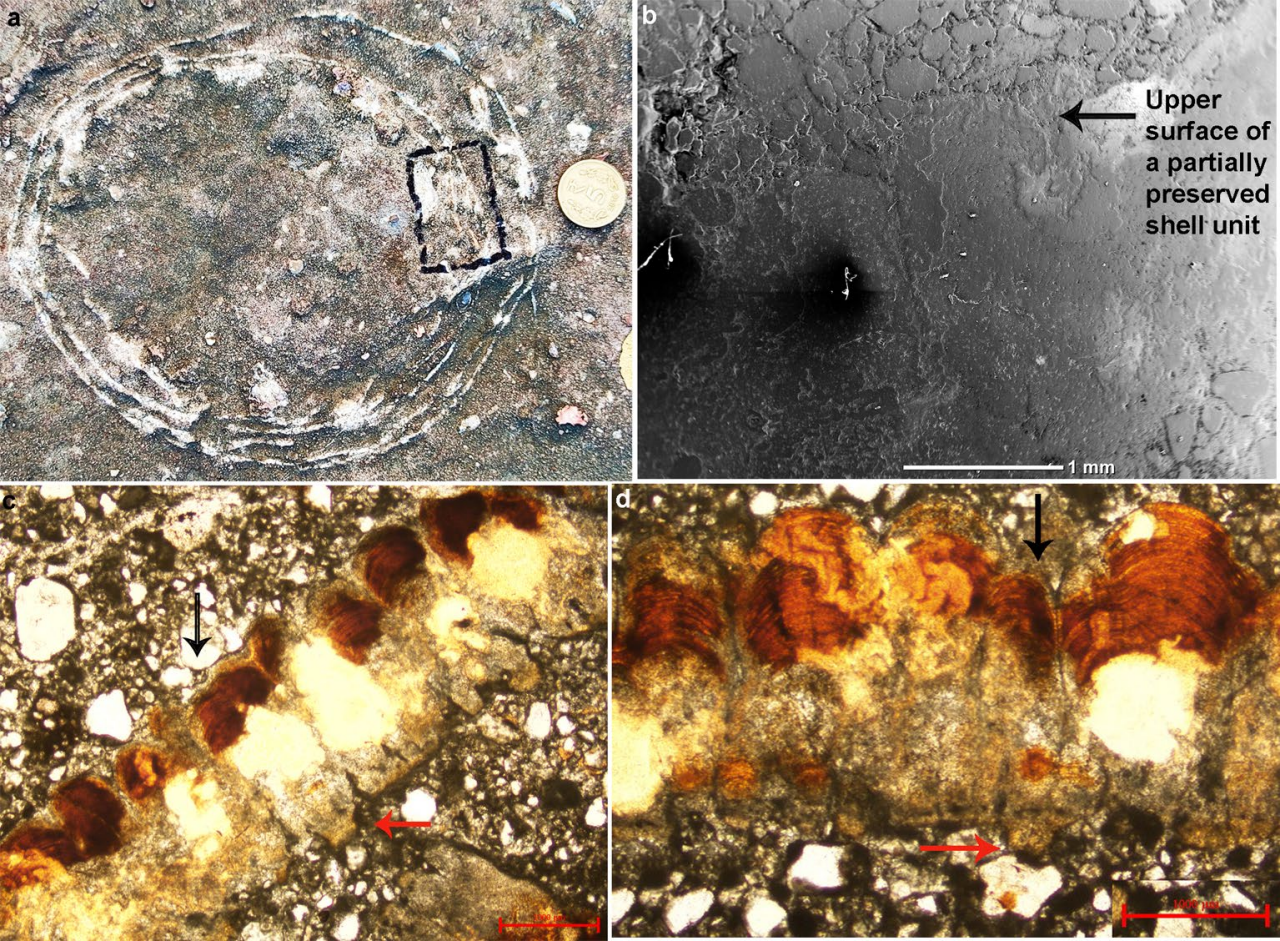
Field photograph and microscopic images of the eggshell from egg number C showing oospecies *Fusioolithus baghensis* characterized by fan-shaped shell units with arching growth lines and basal end cap units. (**a**) The ovum-in-ovo egg with the boxed area showing the region from where the specimen was extracted. (**b**) Radial Scanning Electron Microscope (SEM) photomicrograph of the eggshell showing partially preserved fan-shaped shell unit. (**c**) Photomicrograph of the radial thin section of the eggshell under polarized light microscopy showing partially preserved shell units (see black arrow) and characteristic swollen basal cap units (see red arrow). (**d**) Photomicrograph of the radial thin section of the eggshell under polarized light microscopy exhibiting fused shell units and growth lines (see black arrow) and swollen basal cap (see red arrow).

**Figure 10:**
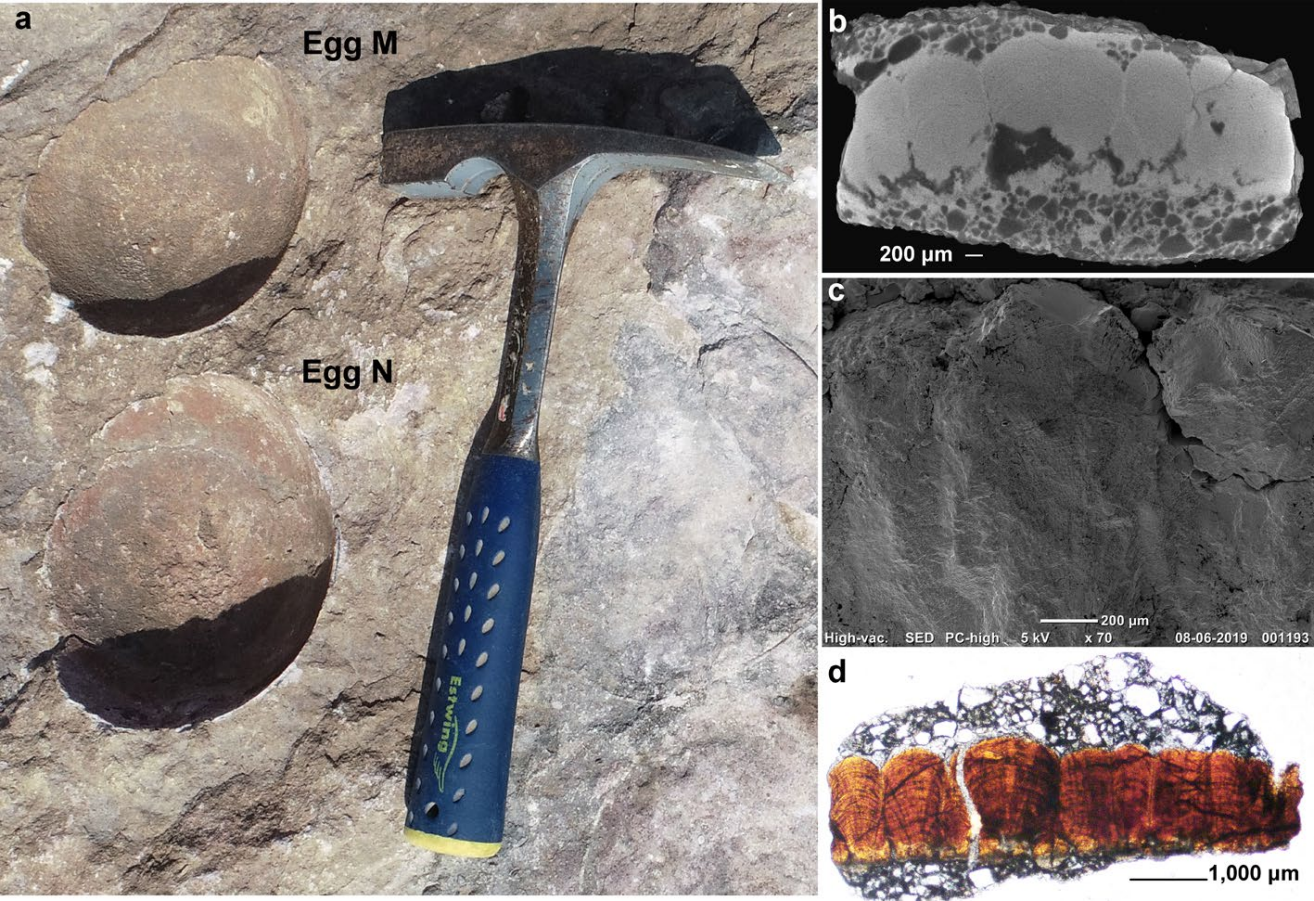
Field photograph and microscopic images of the eggshells from egg number M showing oospecies *Fusioolithus baghensis* characterized by fan-shaped shell units with arching growth lines and basal end cap units. (**a**) Egg numbers M and N present as sub-circular outlines. The eggs were excavated and eggshells were studied for parataxonomic classification. (**b**) X-Ray Microscopy photomicrograph of the eggshell showing fan-shaped shell units progressing upwards into bumpy nodes. Arching growth lines restricted to shell units can also be seen. (**c**) Radial SEM photograph showing curvy growth lines in the middle of the shell unit. (**d**) Radial thin section showing fan-shaped shell units merging with each other in the right segment where growth lines exist as sub-horizontal lines and partially preserved basal end units.

*Material* Eggshell fragments from egg numbers C and M of Figs. 9, 10.

*Description* The eggshells exhibit a tuberculated surface ornamentation consisting of rounded to sub-rounded bumpy nodes separated from each other and showing compactituberculate ornamentation. The average nodal diameter is 0.7 mm and the diameter of the basal end cap unit is 0.2 mm. The average thickness of the shell unit is approximately 2.2 mm and the height/width ratio is 3.1:1. The basic type is spherulitic with discretispherulitic morphotype. In the radial section, the shell units are fan-shaped, wide near the upper part, and fused with a distinct swollen-ended basal cap unit (Figs. 9b–d, 10b–d). The growth lines are moderately arched and show fusion with adjacent growth lines. The margin of intersection of the shell units is vertical to sub-vertical.

*Remarks* Fernández and Khosla^50^ suggested that the basal caps are seen in all three oospecies *M. jabalpurensis*, *M. cylindricus*, and *F. baghensis* but in *F. baghensis,* the basal caps are swollen-ended and not sub-circular. They also mentioned that in *F. baghensis,* the shell units can be both distinct and partially fused and that the growth lines merge and can be horizontal. These features (swollen basal end units, merged growth lines, and both fused and distinct shell units) are observed in both pathologic egg (egg number C) and a nearby normal egg (egg number M) (Figs. 9b–d, 10b–d). This makes us to discount them as the eggs referable either to *M. jabalpurensis *  or *M. cylindricus*.  Based on observed eggshell characteristics we consider them as representing *F. baghensis.* This oospecies has previously been reported from Bagh Cave area, Lametaghat in Madhya Pradesh, Pisdura  in Maharashtra, and Balasinor in Gujarat^49,51^. From Balasinor Quarry, originally it was designated as Type-I^52^ and Kheda Type-A^53^. It was also named as (?)Titanosaurid Type-III from the Lameta Formation of Jabalpur^54^. Vianey-Liaud et al.^55^ made *M. balasinorensis* described by Mohabey^51^ as a junior synonym of *F. baghensis* on the basis of similar shell characteristics. This oospecies has also been documented from the marine lower part of the Upper Cretaceous Kallamedu Formation (Ottakoil)^56^. It is also similar to *M. pseudomamillare* from the Maastrichtian of Les Breguieres, Aix Basin, France^57^, and from the Suterranya locality, Tremp Basin, Southern Pyrenees, Spain, Peru and Bolivia, with French eggs being larger in size^55^. Vianey-Liaud et al.^55^ also made *M. pseudomamillare* a junior synonym of *F. baghensis*. It is similar to type No. 3.2 from Upper Rognacian, Aix Basin, France^57^. This oospecies is also similar to *Patagoolithus salitralensis*^57^ from the Upper Cretaceous strata of Salitral Moreno and Auca Mahuevo, Argentina and *Patagoolithus* was considered as a junior synonym of *F. baghensis*^50,58^. Fernández and Khosla^50^ changed the generic name of these taxa from *Megaloolithus* to *Fusioolithus* on the basis of fused shell units and merged growth lines.

## Discussion

### Pathological nature of titanosaur egg C of nest P7

**Figure 11:**
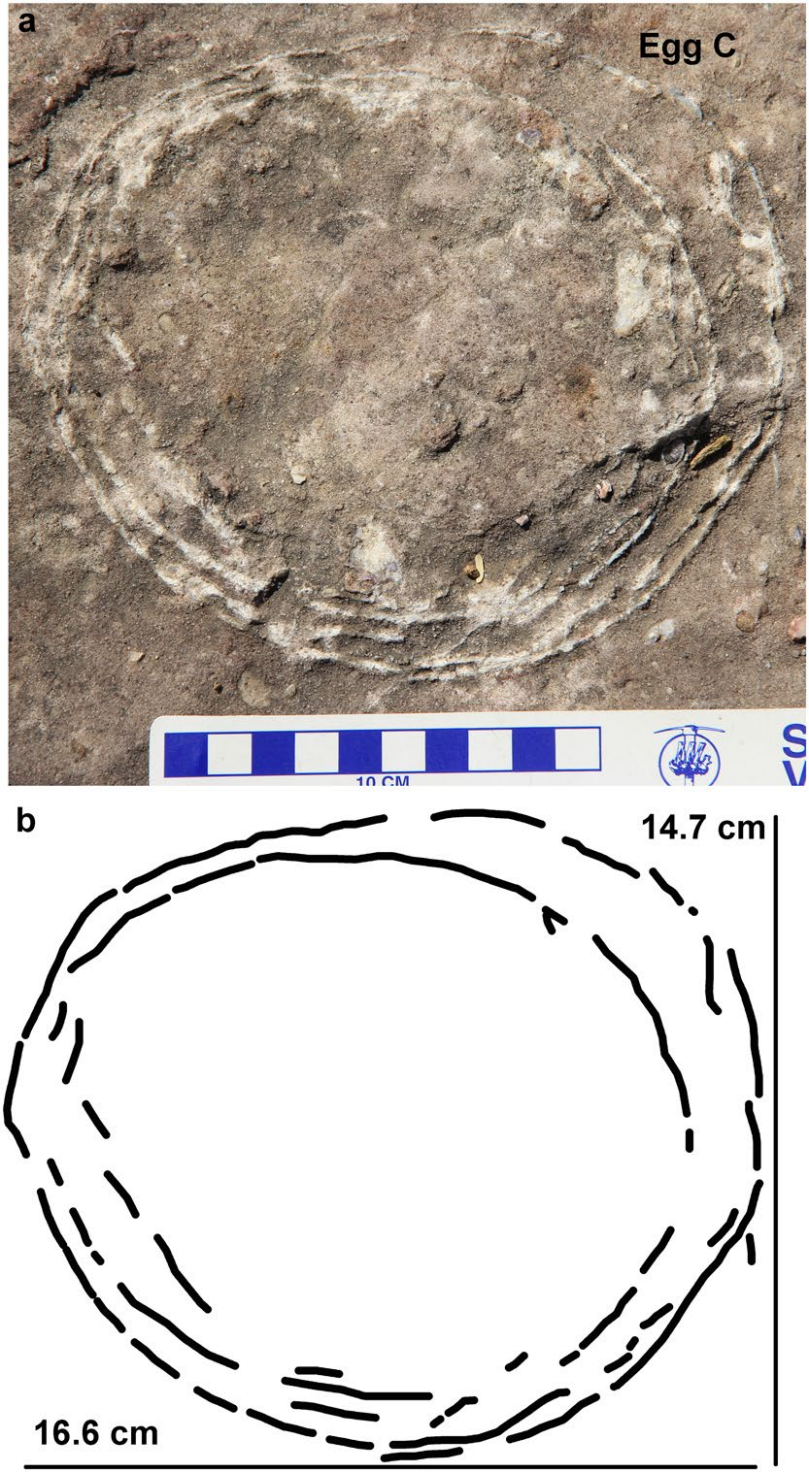
Field photograph and schematic diagram of the pathologic ovum-in-ovo egg (egg number C) documented from the Lameta Formation of village Padlya, Dhar District, M.P., India. Two slightly partially broken circular eggshell outlines can be seen with broken eggshell fragments within. A crescent-shaped gap is characteristically present in the upper right part of the egg (**b** drawn using software Adobe Photoshop CC).

The egg number C of nest P7 shows two circular and partially broken but complete eggshell layers occurring one within the other and with minor eggshell fragments present as curved remnants in between the two layers (Fig. 11). This kind of arrangement of an egg within another egg with a considerable gap in between the two eggshell layers is very similar to the ovum-in-ovo eggs documented in birds^3^ (Fig. 1) and based on this we conclude that egg C represents an ovum-in-ovo pathology. Extant avian eggs with an ovum-in-ovo pathology are known to contain additional yolk and albumen layers between nearly complete inner and outer eggshell layers^18–22^. The gap between the eggshell layers of egg C might have been originally occupied by the yolk prior to fossilization. This mode of preservation is markedly different from that of multi-shelled eggs documented in turtles, geckos, dinosaurs as well as birds. As explained before, in multi-shelled eggs, the mineralised eggshell layers are overlapping and in close contact with each other without any gap separating them.

The two eggshell layers are fairly continuous over much of their perimeter except for some amount of breakage in the upper right side and lower left side of the egg (Fig. 11). The outer layer is relatively more continuous, and the inner eggshell layer exhibits only slight displacement of the broken shells in the left lower part (Fig. 11b). Hatched eggshells accidentally covering the adjacent eggs can be cited for the observed pathology as it was reported in titanosaurid nests of Auca Mahuevo site in Argentina^33^. We discount this explanation for the new find from Padlya as the two calcitic eggshell layers in egg C are not in close contact with each other as is the case with multi-shelled eggshells and eggs of Auca Mahuevo. Rather they are separated from each other by a significant gap and maintain fairly  circular outline of the eggs which is not expected if it was because of accidental and random fall of hatched eggshells. The breakage might have taken place posterior to the burial, possibly due to the deformation of sediments. There is some evidence for deformation of Lameta rocks of Padlya and Dholiya-Raipuria, another dinosaur nesting site located 20 km SW of  Padlya. We have observed a slight displacement of two halves of an egg from  Padlya along its equatorial axis. Similarly, some eggs from Dholiya-Raipuria were stretched sideways into elliptical, partially fractured eggshell layers. We, therefore, conclude that the fracturing within the eggshell layers was caused by the sediment deformation, but the eggshell layers maintained their original circular outline.

### Possible causes for pathology

The presence of multi-shelled eggs and eggshells in a wide range of animals such as turtles, geckos, crocodiles, dinosaurs and birds was interpreted in terms of response of the animals to stress^24,59^. The stress might be the result of several factors, such as sickness, overcrowding leading to competition for space and food resources, scarcity in food resources, floods and droughts, high population density, environmental stress and lack of suitable substrates for nesting^24,32,59^. Pathology has also been attributed to climate  change^23^. More recently, Sellés et al.^60^ discussed the role of climatic and/or environmental changes, dietary behavior change or food type and quality, and ecological competition using stable isotope signatures from normal and abnormal dinosaur eggshells from chronostratigraphically controlled stratigraphic sections of southwestern Europe and ruled out climatic and dietary changes as causes for the development of abnormal eggs. They attributed multi-shelled abnormality in European Maastrichtian eggs to ecological replacement of ornithopod, nodosaurid ankylosaur and titanosaur sauropod communities by hadrosaurid communities at the Early and Late Maastrichtian boundary. No such ecological replacement related stress can be visualised for the development of abnormal egg of the Lameta Formation as titanosaurid was the predominant herbivorous group present in the Late Cretaceous of India. Deccan volcanism, regarded as one of the causes for mass extinctions at the Cretaceous/Palaeogene boundary, was initiated more or less at about the same time as the deposition of the Lameta  Formation. There has been a tremendous decline in the abundance, diversity, and quality of preserved dinosaur fossils from the time of Lameta Formation to slightly younger intertrappean beds (sedimentary beds sandwiched between the volcanic flows). The Lameta dinosaur fauna is represented by a large number of dinosaur bones, teeth, nests, eggs and eggshells belonging to at least three sauropod and five abelisaurid theropod taxa and nine oospecies^58,59^. In comparison, the intertrappean dinosaur fossils are known by rarely occurring teeth and eggshell fragments and in a couple of cases, a few isolated bones which have not been identified below the taxonomic rank of order^61,62^. However, Deccan volcanism cannot be considered as the sole cause for the observed dinosaur egg pathology as only one pathological egg is documented out of 52 nests from the study area. Though we cannot rule out population pressure leading to competition for suitable substrate (palustrine basins of the flood plain) as a possible cause for the observed pathology as the density of nests is high (52 nests per 0.70 square km) in the study area (Padlya), this seems less likely as the pathology is documented in only one egg. Since the number of pathological eggs documented are very limited from the present study area, we believe that it was an individual specific problem and can be attributed to an old or incapacitated individual following injury or sickness or one that underwent significant stress due to jump scare caused by a nearby predator.

### Reproductive biology

Past studies on extant reptiles have demonstrated that increased competition for living space, food resources, and nesting areas greatly affects reproductive functions of the animals leading to the development of pathological eggs^63–65^. Dystocia or prolonged retention of the egg in the uterus has been related to reproductive stress caused by anomalous hormonal function and irregular calcium deposition which leads to the formation of egg pathologies^63–65^. The mechanism of formation of pathologic eggs depends on the reproductive anatomy of the animal, oviduct morphology, and ovulatory pattern^32^. In birds, ovum-in-ovo pathologic egg forms when an egg is pushed back up the oviduct through antiperistaltic waves of muscle contractions where it meets another unshelled egg and then these two subsequently move down to the shell-producing glands to get encased within a second shell layer^3^. If the next ovulation has already taken place, then the egg that has been forced back to more proximal regions of the oviduct through the antiperistaltic waves of muscular contraction receives new layers of membrane, albumen, and/or yolk depending on which oviduct’s part it has been sent back to^66^. The completely shelled individual egg with its yolk may also stay back in the lower part of the oviduct till the unshelled egg comes and through this as well both the eggs can get shelled together^66^. The antiperistaltic waves of muscle contraction in birds have been attributed to stressful conditions^37^. However, the other kind of pathology, the multi-shelled eggshells, was initially documented in turtles, dinosaurs, geckos and possibly crocodilians but was not known in birds. Because of this reason, it was suggested that dinosaurs had possibly a reptilian reproductive system^3,25,31^. But later studies have shown that multi-shelled egg pathology also occurs in birds^32^. This pathology now found in both dinosaur and bird eggs, develops when the egg remains in the shell producing region and as a consequence gets shelled again or when the egg is forced back to the shell gland from its oviposition through muscle contractions and gets shelled^3^.

The bird’s oviduct is divided into five parts, each performing a specialized task during egg’s formation^32^. Unlike birds, turtles have two oviducts, the eggs are simultaneously produced, and the uterus produces both membrane and eggshell^67^. In crocodiles, membrane and eggshell are produced in different regions^32^ in which they share a similar reproductive function with birds^68^. However, different from birds in which one egg of a clutch is ovulated at a time, crocodiles ovulate all the eggs of the clutch simultaneously^32^. Therefore, the reproductive anatomy of crocodiles is somewhat similar to that of birds and distinct from that of other non-archosaurian reptiles (e.g., turtle, lizards) and may represent that of archosaurs^68^.

In view of the occurrence of multi-shelled eggs and eggshells in extant turtles and dinosaurs, it has been hypothesized that dinosaurs had reptile-like reproductive system^32,69^. However, turtles do not provide a good reproductive analogue for dinosaurs as they are non-archosaurians. The titanosaurid nesting behavior which includes partial burial of the clutches as observed in crocodilians, rather than parental incubation as in birds and oviraptorid theropods, has been confirmed by previous water vapor conductance studies^12^. These observations put titanosaur nesting behavior closer to crocodilians. With the addition of the pathologic egg reported here, the presence of both ovum-in-ovo and multi-shelled egg pathologies in both birds and dinosaurs underscores how stress plays a significant role in the development of pathologies irrespective of the kind of reproductive anatomy the animal possesses^32^.

In non-crocodilian reptiles that include turtles, squamates and rhynchocephalians, a single homologous uterus produces both shell membrane and calcitic shell^68,70–72^. In contrast, distinctly different anterior and posterior regions of uterus exist in alligators that function like the isthmus and shell glands of birds in the formation of the shell membrane and calcitic eggshell layer, respectively^68^. Therefore, the reproductive tract of crocodiles and birds is comparable as each part of it performs a specific function unlike that of other reptiles. But in ovulating an entire clutch simultaneously the crocodilian’s ovarian function is similar to other oviparous amniotes, placing them in an intermediate position between amniotes and birds^68^. Palmer and Guillette^68^ suggested that the shared oviductal functional morphology between alligators, crocodiles, and birds is phylogenetically related and the presence of physiological differences, such as ovulatory pattern reduces convergent evolution as a possibility behind shared oviductal functional morphology. Hence crocodiles, though simultaneously ovulate like other reptiles, have separate regions of membrane and eggshell deposition in the uterus like birds, while birds became more specialized using sequential ovulation. This functional divergence formed two branches, one multi-functional uterus and the second representing specialized uterus of the archosaurs.

**Figure 12:**
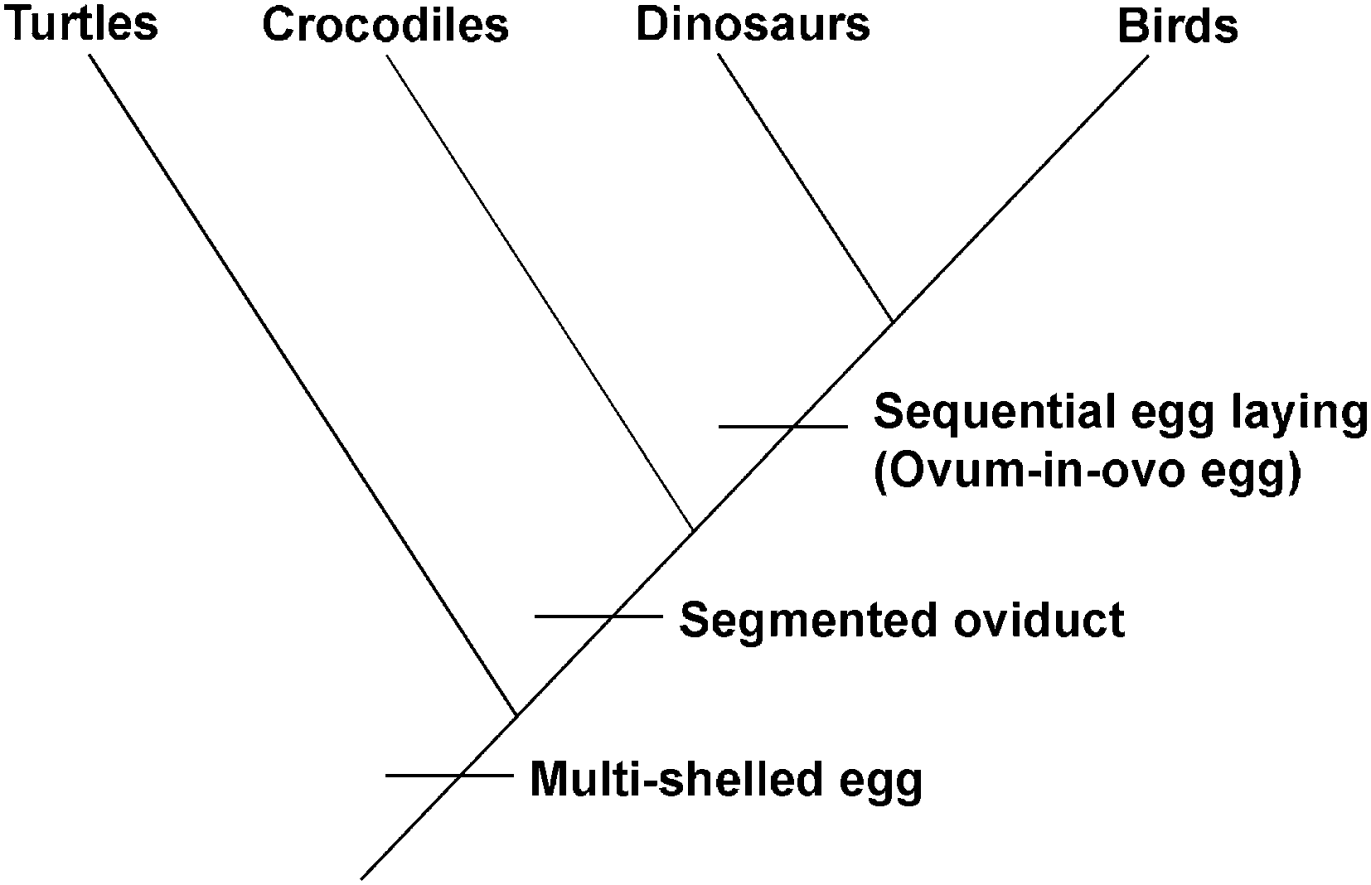
Inferred cladogram showing divergence of dinosaurs from crocodiles on the basis of sequential egg laying (figure drawn using software Adobe Photoshop CC).

If the oviduct of dinosaurs was differentiated like crocodiles and birds, it would have been easy to form the ovum-in-ovo pathologic egg. In birds, when eggs are fully formed, an egg is pushed into cloaca to lay them one by one. For example, in the case of hens, the eggs are not laid during unfavorable conditions^3^. However, the question remains whether titanosaurs laid the eggs sequentially like birds or simultaneously as a clutch like crocodiles and turtles. There were reports in the past about numerous isolated megaloolithid eggs from France that were possibly laid one at a time^46,73^. The discovery of a pathology that has so far been only recorded from birds’ eggs favors the idea that the titanosaurs might have adopted the pattern of sequential laying of eggs like modern birds. The oviduct morphology of birds is capable of producing both types of pathologic eggs as has been observed in both fossil and extant avian eggs. With the previous documentation of multi-shelled eggs from titanosaur nests and the present report of ovum-in-ovo egg, we hypothesize that the titanosaurs possessed a segmented oviduct (similar to birds and crocodiles), sequential ovulation pattern (like modern birds), and nest burial habit (akin to crocodiles on the basis of previous water vapor conductance studies and other physical structures of titanosaur nests). In a phylogenetic context, turtles, crocodiles, dinosaurs and birds all shared the common trait of having multi-shelled eggs. Both turtles and crocodiles have two oviducts, but crocodiles are more derived than turtles in the presence of segmented oviduct and share this derived trait with the birds. Therefore, the segmented oviduct anatomy of crocodiles possibly represents the reproductive anatomy of archosaurs which is intermediate between that of non-archosaurian reptiles and more derived birds. In fact, it has been suggested that the reproductive behaviour of non-avian dinosaurs was intermediate between those of crocodiles and birds as they were derived from a common ancestor ^9^. So far, no ovum-in-ovo pathology was reported in crocodiles. The present discovery of this pathology in titanosaurid sauropod dinosaurs points to possible sequential laying of eggs in dinosaurs as it is the case in birds. Though currently we do not know that dinosaurs had segmented oviductal anatomy, the present find of ovum-in-ovo pathological egg attesting to possible sequential laying of eggs in titanosaurids places dinosaurs close to birds in the cladogram (Fig. 12).

## Conclusions

Ovum-in-ovo and mutli-shelled eggs are the two common pathologies that have been recorded from extinct and extant archosaurs and other amniotes. Current understanding is that ovum-in-ovo pathology is unique to birds and multi-shelled pathology, initially reported in reptiles only, is now regarded common to both reptiles and birds. The present discovery of an ovum-in-ovo pathological egg in a titanosaurid dinosaur nest is the first of its kind in dinosaurs and demonstrates its presence in reptiles as well, specifically in dinosaurs. While several other amniotes have a generalized uterus and laid the eggs simultaneously, the alligators and crocodiles show more similarity with aves by having a specialized segmented uterus while keeping the reptilian mode of egg laying. The presence of ovum-in-ovo pathology from a titanosaur nest supports the idea that the titanosaurs had an oviductal functional morphology similar to birds. This opens up the possibility that titanosaurids might have adapted for sequential laying of eggs. At the moment, it is only a working hypothesis that demands more data to test its validity. Previous water vapor conductance studies and nesting pattern of titanosaurs put them closer to crocodiles than to birds in their reproductive function. Regardless of this, the present find clearly demonstrates that ovum-in-ovo pathology is not unique to birds but also present in dinosaurs. This underscores the fact that sauropod dinosaur reproductive biology is more similar to that of archosaurs (crocodiles, birds) than to non-archosaurian reptiles.

## Methods

Keeping in mind the importance of the documented pathologic egg and to keep intact its shape and structure, only a small eggshell fragment was extracted from the inner eggshell layer of this egg for the purpose of studying microstructure and parataxonomic classification. No other diagnostic body fossil remains of sauropods were found in or around the nest-bearing outcrops in this area. Because of this reason, the sauropod nests and eggs of this area are identified as those of titanosaurids based on the resemblance of eggshell microstructure and ultrastructure to that of eggs with associated embryos or skeletal remains from other parts of the world^7^. The 11 eggs of the nest P7 occur closely with respect to each other and the ultrastructure of egg C and M confirm that the eggs belong to a nest laid by the same animal.

The eggshell fragments were chipped out from the eggs of nest P7 and brought to the Vertebrate Palaeontology Lab of the Department of Geology, University of Delhi (DU). The specimens were catalogued and cleaned in lab under Stereoscopic Binocular Zoom Microscope (Nikon SMZ 745) using brush and fine needles (size 0.2 mm to 1.0 mm). Then they were cleaned in ultrasonic vibrator for a minute followed by cleaning in ethanol. After air-drying photomicrographs of the eggshells were taken under Leica S8 AP0 Stereoscopic Zoom microscope attached with Leica MC120HD digital camera.

For Scanning Electron Microscope photomicrography, the eggshells were mounted on 1 cm diameter aluminium stubs using carbon tape and coated with gold–palladium under vacuum for 10–15 min. They were photographed using JEOL Neoscope 6000 Plus Benchtop SEM with EDS in the Metamorphic Petrology Lab, Department of Geology, University of Delhi. A small fragment of the same specimen studied under SEM was used for making a radial thin section. The eggshell was grinded and polished using carborundum powders and pasted on glass slide using araldite. After the eggshell was firmly fixed to the glass slide, the specimen was grinded away until a thickness of 0.03 mm was achieved and then the surface was polished on a diamond polishing lap. The thin section was studied under Carl Zeiss Axio Imager A1m High Resolution Petrological Microscope in the Vertebrate Palaeontology Lab, DU and Nikon Eclipse 50i Polarizing Microscope with Nikon's Digital Sight DS-U3 camera attachment in Metamorphic Petrology Lab, DU.

## Data Availability

The data is available in the Vertebrate Palaeontology Lab, Department of Geology, University of Delhi.
